# Influence of high fat and different types of carbohydrate diet on energy metabolism in growing mice

**DOI:** 10.20463/jenb.2019.0017

**Published:** 2019-09-30

**Authors:** Nana Chung, Kiwon Lim

**Affiliations:** 1. Department of Physical Education, Sang-ji University, Wonju Republic of Korea; 2. Department of Physical Education, Konkuk University, Seoul Republic of Korea

**Keywords:** High fat-high sucrose, High fat–high starch, Exercise, Energy metabolism

## Abstract

**[Purpose]:**

The purpose of this study was to determine whether different types of carbohydrate diets with or without exercise changes energy metabolism at rest and during exercise.

**[Methods]:**

To minimize differences in food and energy intake between experimental groups, mice were pair-fed. After 1 week of adaptation, 40 male ICR mice (6 weeks old) were randomly divided into four groups: Sta. (high fat + high starch), Scu. (high fat + high sucrose), StaEX. (high fat + high starch + exercise), and SucEX. (high fat + high sucrose + exercise). StaEX. and ScuEX. groups underwent training by running on a treadmill five times a week. After 10 weeks of training, energy metabolism was measured for 24 h and during a 1 h exercise period.

**[Results]:**

The final body weight showed no significant difference between the groups. However, the weight of abdominal tissues (epididymal, perirenal, and mesenteric adipose tissue) in training groups was markedly decreased following 10 weeks of training. Results of all energy metabolism (24 h at rest and during 1 h of exercise) showed no significant interactions between diet and exercise. A brief summary of the results of the energy metabolism is that the metabolism related indicators over 24 h were more affected by the dietary pattern than the exercise but during the 1 h of exercise, training had more effect on energy metabolism than diet.

**[Conclusion]:**

Our findings confirm that: (a) the type of carbohydrates included in the diet influence the metabolic responses over 24 h, (b) training had more effect on energy metabolism than diet during 1 h of exercise, (c) both results; abdominal adipose tissue weight and fat oxidation during exercise are suggestive for a beneficial effect of moderate physical activity on weight maintenance.

## INTRODUCTION

Child obesity has become a significant health issue in Korea. Along with the adaptation to a Western diet over the past decades, the dietary patterns of the Korean population, especially Korean children and adolescents, have gradually been changing. The Western diet includes not only a diet high in processed or fast foods, but also physical inactivity^1^. Nowadays, many children spend a great deal of time being inactive. According to statistics, the average child spends approximately 4 hours each day watching television^2^. In addition, they spend most of their spare time playing video or computer games and cellphone. This can further increase the physical inactivity in children. The other characteristic of a Western diet is the high dietary lipid and sugar content present in processed foods^3^. Although the impact of specific macronutrients on health is highly debated, it is evident that diets rich in fat and carbohydrates, with or without exercise, result in obesity. Because of their palatability and higher energy density than that of other macronutrients, high fat diets may lead to excessive energy intake and cumulative positive fat balance. Moreover, because of the lower dietary induced thermogenesis, high fat diets may also decrease energy expenditure^4^^,^^5^. There is no apparent autoregulation between fat intake and fat oxidation. Fat oxidation levels are changed by the intake of other macronutrients^6^. On the other hand, carbohydrate oxidation shows strong autoregulation with carbohydrate intake because the capacity of glycogen storage is limited. The diet-induced thermogenesis associated with high carbohydrate diets would be higher than that associated with high fat diets because of glycogen storage and glucose stimulation of the sympathetic nervous system^7-10^. The types of carbohydrate may also influence metabolic responses such as thermogenesis and substrate utilization because the nutritional properties of carbohydrates depend on their rate and extent of digestion and absorption in the small intestine^11^.

In this study, we used two types of carbohydrates, sucrose and starch. Sucrose, the scientific name for sugar, is a disaccharide, which is a combination of two monosaccharides^12^. Sucrose is often combined with other sugars in foods such as sweets and beverages. It can also be hidden in many processed foods. Starches, sometimes called complex carbohydrates, are polysaccharides found in rice, the staple food of Koreans, as well as breads, potatoes, and corn. Hence, the body has to first break down the links between each sugar subunit in order to use the energy of the sugar molecules in the starch. This causes rapid increases in blood sugar levels, which stimulates the release of a large amount of insulin. Insulin draws sugar into the cells to be stored as glycogen, leading to a subsequent drop in blood sugar, which often leads to further consumption of carbohydrates. This phenomenon is called 'blood sugar roller-coaster'^11^. This triggers insulin resistance, where the cells become fatigued from constant high insulin signaling and no longer respond adequately to its release. Poor blood sugar regulation also stimulates the release of stress hormones, such as cortisol, which can contribute to weight gain as well as hypoglycemia^13^. In this sense, sucrose and starch may also have different effects on energy metabolism.

Although there is increasing interest in understanding the relationships between different types of dietary carbohydrates and appetite regulation, energy metabolism, body weight, and body composition, little is known about the impact of the consumption of different types of carbohydrates on metabolic responses, at rest and during exercise, in childhood. Most previous studies have focused only on the effects of diet^14-17^ and exercise^18-20^ on the changes in energy metabolism at rest and during exercise. There is limited information on the effects of simultaneous consumption of high fat and different types of carbohydrates (sucrose or starch), with or without exercise, on body weight, fat accumulation, and energy metabolism. Hence, the purpose of this investigation was to study the change in the fuels oxidized at rest and during exercise on a high fat-high sucrose and high fat-high starch diet, with or without exercise, in growing mice as one possible method to address child obesity.

## METHODS

### Animals and treatments

Prospective clinical studies on a high fat-high sucrose diet would be unethical because of the known detrimental metabolic consequences of this diet in humans. Therefore, we employed an animal model to examine the effect of a high-fat diet combined with different types of carbohydrates on energy metabolism. Five-week-old male ICR mice (n = 40) were purchased from Orient Rio Company (Seongnam, Korea). All mice were kept in a specific pathogen-free (SPF) environment (humidity; 50% and temperature 23 ± 1 °C) and housed in conventional cages (n = 5 per cage) under a standard 12 h light/dark cycle. Food intake and body weight were measured daily for 10 weeks.

### Experimental design

During the one-week adaptation period, general clinical symptoms were observed and mice were selected and randomized according to the weight range. After adaptation, mice (6 weeks old) were randomly divided into four groups: Sta. (high fat + high starch), Scu. (high fat + high sucrose), StaEX. (high fat + high starch + exercise), and SucEX. (high fat + high sucrose + exercise). StaEX. and ScuEX. groups underwent training by running on a treadmill five times a week. After 10 weeks of training, energy metabolism was measured for 24 h at rest and during 1 h of exercise.

All experimental procedures were approved by the Animal Experiment Research Center of Konkuk University and Ethics Committee of the Konkuk University Institutional Animal Care and Use Committee (Permit Number: KU17114).

### Composition of the diets

We designed studies to test the metabolic flexibility of mice to adapt to high fat and high sucrose or high starch diets through changes in substrate utilization. To clarify the influence of different types of carbohydrates on energy metabolism, mice were fed a high sucrose or a high starch diet containing identical amounts of proteins and lipids. Diets were matched for macronutrient and micronutrient content and the energy content of the two diets was identical (Table. 1). To minimize differences in food and energy intake between experimental groups, mice were pair-fed and the high fat–high starch diet was served as a baseline.

**Table 1.** Composition of the diets

	HF+Hsta gm%	kcal%	HF+Hscu gm%	kcal%
Corn starch	40	39	5	39
Sucrose	5		40	
Casein	24	21	24	21
Corn oil	21	40	21	40
Total	90	100	90	100
Density(kcal/g)	4.7		4.7	
Vitamin mix.	1		1	
Mineral mix.	4		4	
Cellulose	5		5	

### Training method

The exercise group used a treadmill for small animals. All mice in exercise groups (n = 20) were first introduced to running for 3 days. The mice were then trained five times per week for 10 weeks by gradually ramping up the speed and time under the following training conditions: 15 m/min, 8° slope, 50 min/day for the first two weeks, then ramped up to 25 m/min, 8° slope, 60 min/day, at approximately 70–75% of the maximum oxygen uptake for the last four weeks.^21-24^

**Figure 1.**
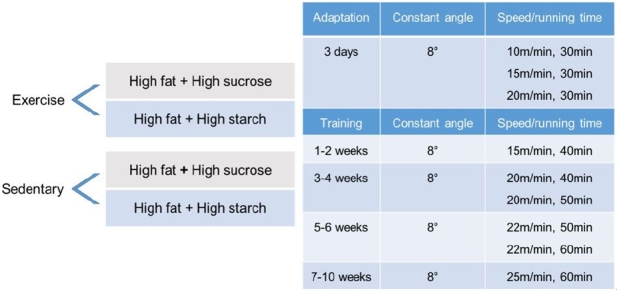
Experimental group design and training program.

Details of the experimental group design and training program are shown in Figure. 1. To keep the mice running, the grid at the back of the treadmill delivered a mild electric foot shock. Mice in the sedentary group (n = 20) remained in their cages for the duration of the 10-week training program.

### Resting metabolic rate (RMR) analysis 

One week before the end of the experiment, the RMR was measured by indirect calorimetry, using an open-circuit device based on methods reported in previous studies^25^^,^^26^ for 24 h stabilization. Two hours before the start of the measurement, the mice were placed in a metabolic chamber with a volume of approximately 1 L to reduce stress.^26^^,^^27^ The flow rate was kept constant at 1.2 L/min^26^. During measurement, data were recorded for each diet according to their group and water was freely available.

### Energy metabolism alterations during exercise

After 10 weeks of training, energy metabolism was measured for 1 h using exercise metabolism chambers^23^^,^^28^ under the training conditions of 18 m/min, 8° slope, and 50–55% of maximal oxygen uptake. Mice were placed in exercise metabolism chambers for adaptation 2 h before the measurement.

### Statistical analysis

Statistical analyses were performed with SPSS 23.0 for windows (IBM Corp., Armonk, USA). Values are means ± standard error (SE) of the indicated number of experiments. A two-way analysis of variance (ANOVA) method was applied to determine the interaction and main effect with diet and exercise. A Tukey’s post-hoc analysis was conducted if significant interactions or main effects were obtained. A priori, the level of significance was set at 0.05.

## RESULTS

### Changes in body weight and abdominal adipose tissue weight

**Figure 2.**
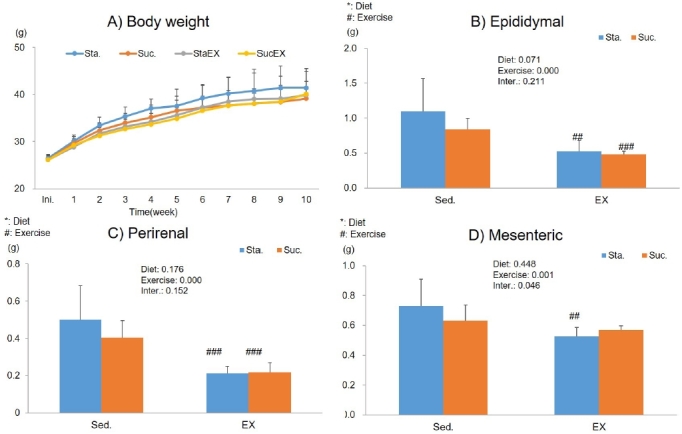
Influence of a high fat and different types of carbohydrate diet or/and exercise on body weight and abdominal adipose tissue weight. (A) Body weight (B) Epididymal adipose tissue weight (C) Perirenal adipose tissue weight (D) Mesenteric adipose tissue weight. Data are presented as means ± SE (n = 10 per group). Values in the same row not sharing the same superscript differ significantly (^#^P < 0.05, ^##^P < 0.01, ^###^P < 0.001).

To clarify the influence of a high fat diet with different types of carbohydrates and/or exercise on basic metabolism in mice, the data of mice at Sta. (HF + Hsta), or Scu. (HF + Hscu) with/without exercise for 10 weeks were compared. Figure 2 shows the results of body weight and change in epididymal, perirenal, and mesenteric adipose tissue weight. At the onset of the study, there were no differences in body weight between the four groups. Body weight of mice increased progressively throughout the study, but unexpectedly, there were no significant differences in final body weight between the groups (Fig. 2A). 

Next, we determined whether diet and exercise changed the weight of abdominal tissues at the end of the experimental period. The weight of epididymal and perirenal adipose tissue were significantly decreased in the exercise groups (epididymal; StaEX, P = 0.004 and SucEX, P = 0.000 Fig. 2C, perirenal; StaEX, P = 0.004 and SucEX, P = 0.000, Fig. 2C). The weight of mesenteric adipose tissue decreased only in the Sta. diet in the exercise group (P = 0.001, Fig. 2D). 

### Energy metabolism over 24 hours

**Figure 3.**
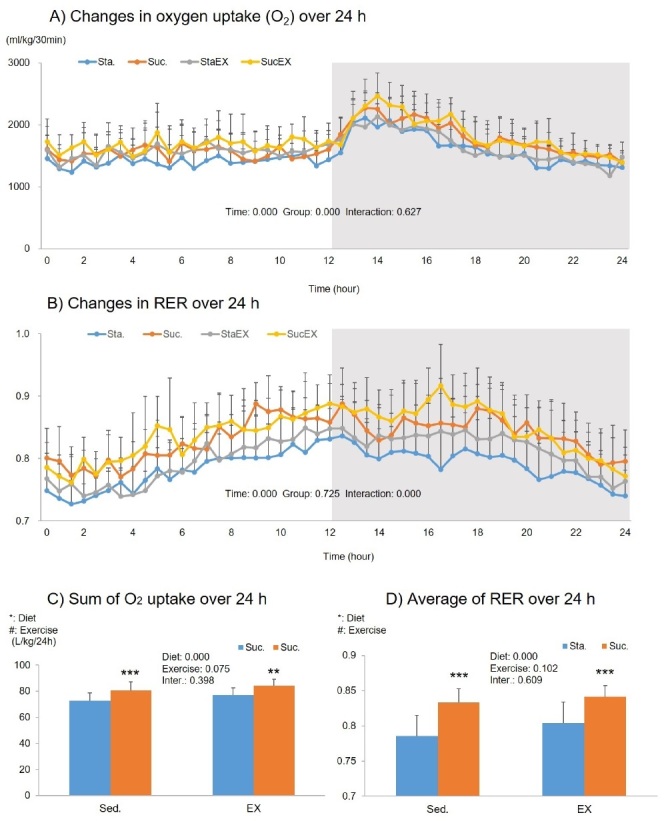
Influence of a high fat diet with different types of carbohydrates and/or exercise on oxygen uptake and respiratory changes ratio (RER) over 24 h. A) Changes in oxygen uptake over 24 h. B) Changes in RER over 24 h. C) Sum of oxygen uptake over 24 h. D) Average of RER over 24 h. Data are presented as means ± SE (n = 10 per group). Values in the same row not sharing the same superscript differ significantly (*P < 0.05, **P < 0.01, ***P < 0.001).

The two-way repeated measures ANOVA of the oxygen uptake showed a significant time (P = 0.000) and group (P = 0.000) related effect, but no group-time interaction (P = 0.627, Fig. 3A). The sum of the oxygen uptake over 24 h showed a significant diet effect (P = 0.000). This indicates that oxygen uptake increased in the Suc. diet groups (Suc. P = 0.000 and SucEX, P = 0.002, Fig. 3C).

The two-way repeated measures ANOVA for RER showed a significant time effect (P = 0.000) and a group-time interaction (P = 0.000), but no group effect (P = 0.725, Fig. 3B). Similar to oxygen uptake, we observed significant diet effects on average RER during 24 h. There was a highly significant increase in the average RER in the Suc. diet groups (Suc. P = 0.000 and SucEX, P = 0.000, Fig. 3D).

**Figure 4.**
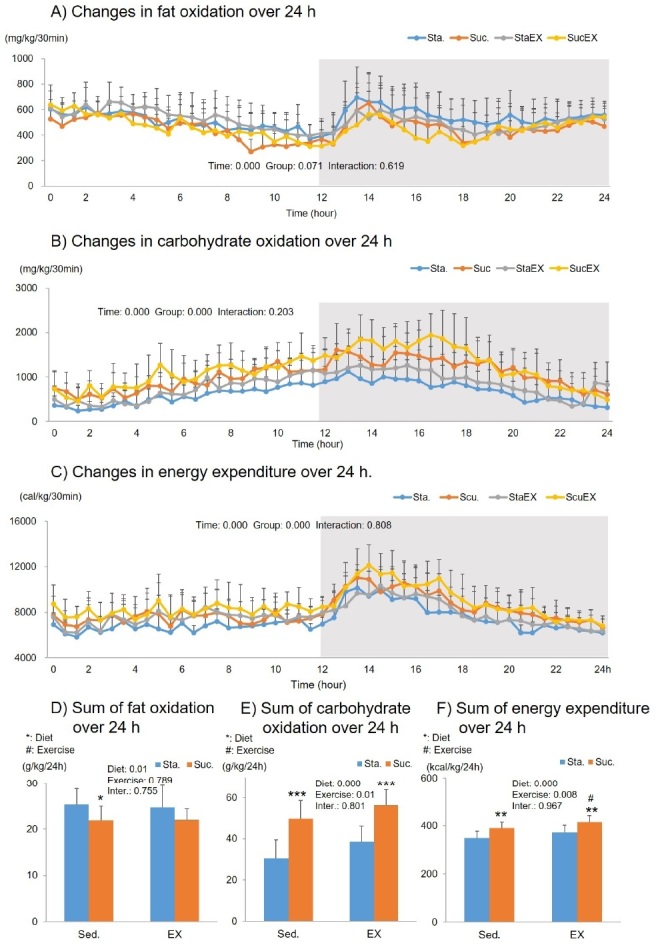
Influence of a high fat diet with different types of carbohydrates and/or exercise on fat oxidation, carbohydrate oxidation, and energy expenditure over 24 h. A) Changes in fat oxidation over 24 h. B) Changes in carbohydrate oxidation over 24 h. C) Changes in energy expenditure over 24 h. D) Sum of fat oxidation over 24 h. E) Sum of carbohydrate oxidation over 24 h. F) Sum of energy expenditure over 24 h. Data are presented as means ± SE (n = 10 per group). Values in the same row not sharing the same superscript differ significantly (*^/#P^ < 0.05, **P < 0.01, ***P < 0.001).

The two-way repeated measures ANOVA for fat oxidation showed a significant time (P = 0.000) related effect, but no group effect (P = 0.071) or group-time interaction (P = 0.619, Fig. 4A). The sum of the fat oxidation for 24 h, was significantly affected by diet (P = 0.01). The sum of fat oxidation in the Suc. diet without exercise group (Scu. P = 0.031) was lower than that of the Suc. diet with exercise group (P = 0.031 and P = 0.128, respectively) (Fig. 4D).

The two-way repeated measures ANOVA for carbohydrate oxidation showed a significant time (P = 0.000) and group related effect (P = 0.000), but no group-time interaction (P = 0.203, Fig. 4B). The sum of the carbohydrate oxidation for 24 h, was significantly affected by diet (P = 0.000) and exercise (P = 0.001). The post-hoc analyses showed that the increase in total carbohydrate oxidation was significantly higher in the Suc. diet than in the Sta. diet (Sta. P = 0.000 and StaEX. P = 0.000, Fig. 4E).

The two-way repeated measures ANOVA for total energy expenditure showed a significant time (P = 0.000) and group (P = 0.000) related effect, but no group-time interaction (P = 0.808, Fig. 4C). The sum of the energy expenditure for 24 h was significantly affected by diet (P = 0.000) and exercise (P = 0.008). Total energy expenditure was higher in the Suc. diet (Sta. vs. Scu. P = 0.002 and StaEX. Vs. ScuEx. P = 0.003), and the Suc. diet with exercise group had significantly higher energy expenditure than that of the Suc. diet without exercise group (P = 0.032 Fig. 4F).

### Energy metabolism during exercise

**Figure 5.**
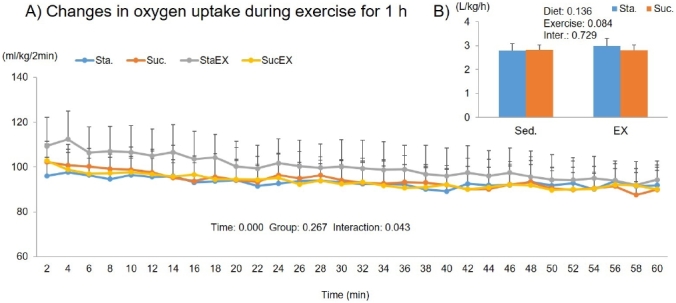
Influence of a high fat diet with different types of carbohydrates and/or exercise on oxygen uptake during exercise. A) Changes in oxygen uptake during exercise for 1 h. B) Sum of oxygen uptake during exercise for 1 h. Data are presented as means ± SE (n = 10 per group).

The two-way repeated measures ANOVA for oxygen uptake during a 1 h exercise period showed a signiﬁcant time effect (P = 0.000) and a group-time interaction (P = 0.043), but no significant group effect (P = 0.267, Fig. 5A). The sum of oxygen uptake during 1 h of exercise was not affected by diet composition (P = 0.136) and exercise (P = 0.084). There were no differences among the groups (Fig. 5B).

**Figure 6.**
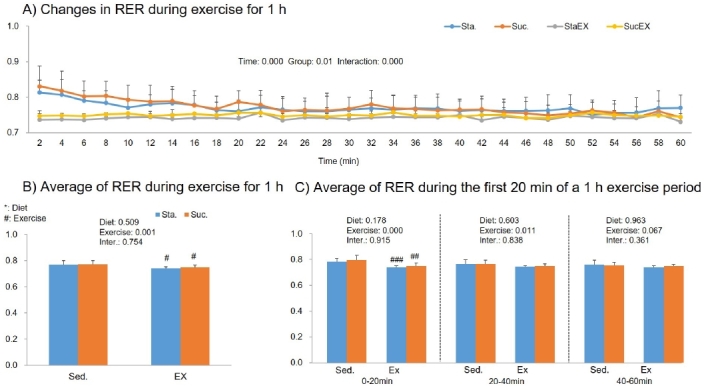
Influence of a high fat diet with different types of carbohydrates and/or exercise on respiratory changes ratio (RER) during exercise. A) Changes in RER during exercise for 1 h. B) Average of RER during exercise for 1 h. C) Average of RER during the first 20 min of a 1 h exercise period. Data are presented as means ± SE (n = 10 per group). Values in the same row not sharing the same superscript differ significantly (^#^P < 0.05, ^##^P < 0.01, ^###^P < 0.001).

The two-way repeated measures ANOVA for RER during 1 h of exercise showed signiﬁcant time (P = 0.000) and group effects (P = 0.01) and a group-time interaction (P = 0.000, Fig. 6A). In the average of RER during 1 h of exercise, we observed a significant exercise effect (P = 0.001, Fig. 6B). The average RER was lower in exercise groups (StaEx. P = 0.032 and ScuEx. P = 0.027). In addition, we analyzed this again, measuring every 20 min, and found that exercise training decreased RER during 1 h of exercise in the first 20 min, regardless of diet composition (StaEX. P = 0.000 and ScuEx, P = 0.002, Fig. 6C).

**Figure 7.**
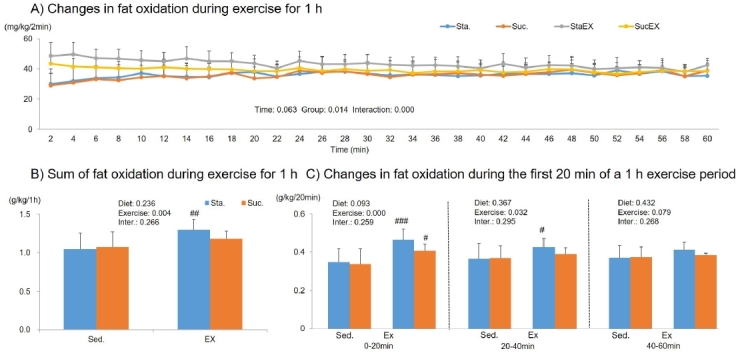
Influence of a high fat diet with different types of carbohydrates and/or exercise on fat oxidation during exercise. A) Changes in fat oxidation during exercise for 1 h. B) Sum of fat oxidation during exercise for 1 h. C) Changes in fat oxidation during the first 20 min of a 1 h exercise period. Data are presented as means ± SE (n = 10 per group). Values in the same row not sharing the same superscript differ significantly (^#^P < 0.05, ^##^P < 0.01, ^###^P < 0.001).

The two-way repeated measures ANOVA for fat oxidation during 1 h of exercise showed a signiﬁcant group effect (P = 0.014) and a group-time interaction (P = 0.000), but no time effect (P = 0.063, Fig. 7A). Similar to RER, only exercise had an effect on fat oxidation (P = 0.004). The sum of fat oxidation increased in the Sta. with exercise group (StaEX. P = 0.004, ScuEX. P = 0.164, Fig. 7B). However, when the 1 h exercise period was split into 20 min intervals, the results showed that, during the first 20 min, training increased fat oxidation regardless of diet (StaEX. P = 0.000, ScuEX. P = 0.037). However, the exercise effect was valid for up to 40 min only in the Sta. with exercise group (P = 0.025, Fig. 7C).

**Figure 8.**
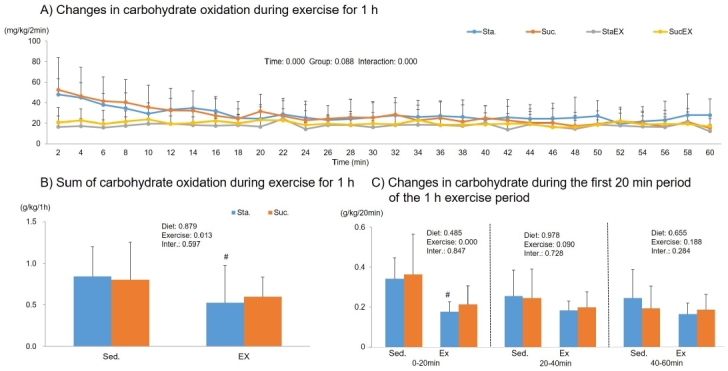
Influence of a high fat diet with different types of carbohydrates and/or exercise on carbohydrate oxidation during exercise. A) Changes in carbohydrate oxidation during exercise for 1 h. B) Sum of carbohydrate oxidation during exercise for 1 h. C) Changes in carbohydrate during the first 20 min period of the 1 h exercise period. Data are presented as means ± SE (n = 10 per group). (^#^P < 0.05)

The two-way repeated measures ANOVA for carbohydrate oxidation during 1 h of exercise showed a signiﬁcant time effect (P = 0.000) and a group-time interaction (P = 0.000), but no group effect (P = 0.088, Fig. 8A). The sum of carbohydrate oxidation during 1 h of exercise was significantly affected by exercise (P = 0.013). The post-hoc analyses showed that total carbohydrate oxidation increased significantly in the Sta. diet with exercise group (P = 0.043, Fig. 8B) and this occurs in the first 20 min of exercise (P = 0.031, Fig. 8C).

**Figure 9.**
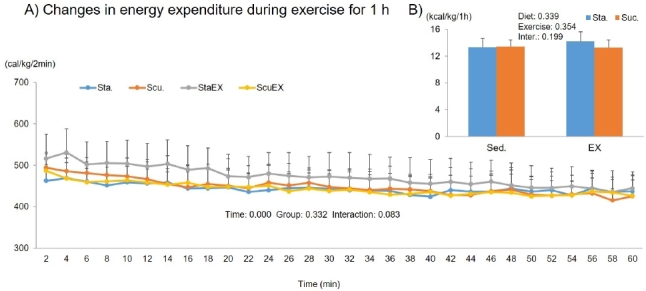
Influence of a high fat diet with different types of carbohydrates and/or exercise on total energy expenditure during exercise. A) Changes in energy expenditure during exercise for 1 h. B) Sum of energy expenditure during exercise for 1 h. Data are presented as means ± SE (n = 10 per group).

The two-way repeated measures ANOVA for energy expenditure showed only a significant time effect (P = 0.000), there were no group effects (P = 0.332) or a group-time interaction (P = 0.083, Fig. 9A). Moreover, the sum of energy expenditure during 1 h of exercise was not affected by diet composition (P = 0.339) or exercise (P = 0.354). There was no difference among the groups (Fig. 9B).

## DISCUSSION

Along with the adaptation to a Western lifestyle over the past decades, the dietary patterns of Korean children and adolescents have been gradually changing. A Western lifestyle includes not only a diet rich in lipids and sugar, but also physical inactivity. Although there is strong evidence that the amount and type of fat in the diet can have strong effects on metabolism, the type of carbohydrates influencing metabolic responses in a diet is also of great interest. Hence, the long-term goal of this study was to understand the relationship between type of carbohydrates, weight, and energy metabolism, and to prevent childhood obesity and related diseases. To achieve this, we used an open circuit calorimetry system to investigate the influence of a high fat diet with different types of carbohydrates (sucrose or starch) with/without exercise on energy metabolism at rest and during 1 h of exercise in mice.

Our results showed that high sucrose did not cause greater obesity than starch. Unexpectedly, there were no significant differences between the groups in the final body weights. It is consistent with a previous study by Sakamoto et al., (2012) that demonstrated no change in body weight following similar, prolonged periods of high sucrose feeding^29^. A previous study on energy balance by Dulloo et al. (1985) indicated that differences in energy balance between sucrose and starch are small^30^. Indeed, several reviews suggest that there is no evidence that an ad libitum diet with sucrose causes more weight gain than a diet with starch when total carbohydrate intake is the same^31^^,^^32^. On the other hand, some studies showed weight gain^7^^,^^33^ and an overall systematic review suggested a positive influence of starch on weight loss^34^. However, it is not clear what caused the differences between these two types of diets. Only a few studies have directly compared the effect of dietary intake of sucrose and starch on energy metabolism. According to the results of the study by Maekawaa et al., (2017), high sucrose-fed mice displayed decreased body weight compared with mice on a diet of normal chow or high starch after 14 weeks of feeding^35^. They explained that this was due to increased energy expenditure. In addition, Raben et al. (1997) compared the effect of an ad libitum high sucrose, high starch, and high fat diet in lean or post-obese women on energy expenditure^36^. After 14 days, the 24 h energy expenditure was significantly increased on the sucrose diet compared with that of the other diets. 

We then investigated the effect of the sucrose diet on energy metabolism using indirect calorimetry to compare each mouse after 10 weeks of experimental treatment. As expected, mice on a high fat–high sucrose diet consistently displayed a higher oxygen uptake and RER, indicating an increase in carbohydrate oxidation at rest compared with that of the Sta. diet. This implies both an initial switch to carbohydrates as the major energy source and continued adaptation to further increase carbohydrate use. However, in contrast to the results of the present study, Burchfield et al. (2018) showed that RER and oxygen uptake of mice on a high fat–high sucrose diet decreased with increasing time on the diet^37^.

Fat oxidation in the Scu. diet was lower than that in the Sta. diet. However, there were no differences between the Suc. diet with exercise and the Sta. diet groups. This indicates that exercise helps to maintain the fat oxidation capacity in the Scu. diet. Although the differences did not result in significant weight reduction, total energy expenditure was increased in the Suc. diet with or without exercise. Small differences of just a few kcal/day can lead to substantial differences in body weight in the long term. Taken together, our findings indicate that energy metabolism over 24 h was more influenced by diet than by exercise.

The expression of some genes controlling energy homeostasis are regulated by epigenetic mechanisms that may play a role in body weight regulation. It is well recognized that sucrose stimulates the sympathetic nervous system and leads to an increase in brown adipose tissue (BAT) activity and mass^7^^,^^38^^,^^39^. However, the pathway by which sucrose affects BAT activity and the effects of a chronic high sucrose diet on BAT, which plays an important role in energy expenditure, are not well understood. In addition, body weight and glucose metabolism is regulated by various hormones such as glucagon-like peptide 1 (GLP-1) and fibroblast growth factor 21 (FGF21), which contribute to the reduction in body weight by increasing energy expenditure^35^^,^^40^^,^^41^. However, the expression and action of GLP-1 and FGF21 in mice chronically fed a high fat-high sucrose diet have not been fully investigated^35^. Thus, further studies are required to confirm the optimal amount and type of dietary carbohydrates for energy metabolism.

During 1 h of exercise, we did not find any changes in oxygen uptake and energy expenditure in any of the experimental groups. In agreement with our results, most previous studies^42-44^ have not found an effect of diet composition on total energy expenditure if energy intake was fixed. However, exercise had a significant effect on RER, fat oxidation, and carbohydrate oxidation. The average RER was lower in exercise groups; in particular, we found that the initial 20 min RER phase during the 1 h exercise period was significantly lower in exercised groups regardless of diet composition. Substrate utilization is largely driven by the availability of substrate. Excess carbohydrate intake promotes its own oxidation by stimulating the cellular uptake and oxidation of glucose during exercise, while decreasing the fat availability and oxidation^45^. Exercise, however, had a positive effect on fat oxidation during the 1 h exercise period. This effect was seen for up to 40 min in the Sta. diet group. Taken together, these results indicate that energy metabolism during exercise was more influenced by exercise than diet. In our experiments, mice were made to exercise for approximately 70–75% of maximum oxygen uptake. However, high intensity exercise can also mediate stress related hormones, such as glucocorticoids, cortisol, and catecholamine^13^ that trigger the release of glucose stored as glycogen into the bloodstream, leading to high blood sugar levels, an insulin response, and therefore, metabolic diseases^46^.

A limitation of this study is that the above results alone cannot prove that sucrose is not harmful. The greatest difference between sucrose and starch is probably the blood glucose and insulin response, and there may also be differences in some genes controlling energy homeostasis. Hence, future research should focus on the influence of high fat diets with different types of carbohydrates and/or exercise on more functional aspects, including glucose, insulin in blood, and UCP1, PGC1a, GLP-1, and FGF21 in the adipose tissue.

Our results showed that high sucrose did not cause greater obesity than starch. The weight of adipose tissue decreased markedly in exercise groups. Dietary sucrose increased energy expenditure during a 24 h period by increasing carbohydrate oxidation. Long-term exercise training increased fat oxidation during 1 h of exercise, regardless of diet. On the basis of these results, our study demonstrates that: (a) the type of carbohydrates included in the diet influence the metabolic responses for 24 h, (b) training had a larger effect on energy metabolism than diet during 1 h of exercise, (c) both abdominal adipose tissue weight and fat oxidation during exercise for 1 h showed the beneficial effects of moderate physical activity on weight maintenance. 
